# A Prospective Study of the Impact of Severe Childhood Deprivation on Brain White Matter in Adult Adoptees: Widespread Localized Reductions in Volume But Unaffected Microstructural Organization

**DOI:** 10.1523/ENEURO.0188-22.2022

**Published:** 2022-11-03

**Authors:** Nuria K. Mackes, Mitul A. Mehta, Ahmad Beyh, Richard O. Nkrumah, Dennis Golm, Sagari Sarkar, Graeme Fairchild, Flavio Dell’Acqua, Edmund J. S. Sonuga-Barke, on behalf of the ERA Young Adult Follow-Up Team

**Affiliations:** 1 Department of Psychological Medicine, Institute of Psychiatry, Psychology and Neuroscience, King’s College London, London SE5 8AF, United Kingdom; 2 Department of Neuroimaging, Institute of Psychiatry, Psychology and Neuroscience, King’s College London, London SE5 8AF, United Kingdom; 3 Department of Forensic & Neurodevelopmental Sciences. Institute of Psychiatry, Psychology and Neuroscience, King’s College London, London SE5 8AF, United Kingdom; 4 Department of Neuroimaging, Central Institute of Mental Health Mannheim, Medical Faculty Mannheim, Heidelberg University, Mannheim J5 68159, Germany; 5 Centre for Innovation in Mental Health, School of Psychology, University of Southampton, Southampton SO17 1PS, United Kingdom; 6 Cognitive Neuroscience & Neuropsychiatry, Great Ormond Street Institute of Child Health, University College London, London WC1N 1EH, United Kingdom; 7 Department of Psychology, University of Bath, Bath BA2 7AY, United Kingdom; 8 Department of Child and Adolescent Psychiatry, Institute of Psychiatry, Psychology and Neuroscience, King’s College London, London SE5 8AF, United Kingdom; 9 Department of Child & Adolescent Psychiatry, Aarhus University, Aarhus 8000, Denmark

**Keywords:** brain imaging, early adversity, institutional deprivation, plasticity, tractography, white matter structure

## Abstract

Early childhood neglect can impact brain development across the lifespan. Using voxel-based approaches we recently reported that severe and time-limited institutional deprivation in early childhood was linked to substantial reductions in total brain volume in adulthood, >20 years later. Here, we extend this analysis to explore deprivation-related regional white matter volume and microstructural organization using diffusion-based techniques. A combination of tensor-based morphometry (TBM) analysis and tractography was conducted on diffusion-weighted imaging (DWI) data from 59 young adults who spent between 3 and 41 months in the severely depriving Romanian institutions of the 1980s before being adopted into United Kingdom families, and 20 nondeprived age-matched United Kingdom controls. Independent of total volume, institutional deprivation was associated with smaller volumes in localized regions across a range of white matter tracts including (1) long-ranging association fibers such as bilateral inferior longitudinal fasciculus (ILF), bilateral inferior fronto-occipital fasciculus (IFOF), left superior longitudinal fasciculi (SLFs), and left arcuate fasciculus; (2) tracts of the limbic circuitry including fornix and cingulum; and (3) projection fibers with the corticospinal tract particularly affected. Tractographic analysis found no evidence of altered microstructural organization of any tract in terms of hindrance modulated orientational anisotropy (HMOA), fractional anisotropy (FA), or mean diffusivity (MD). We provide further evidence for the effects of early neglect on brain development and their persistence in adulthood despite many years of environmental enrichment associated with successful adoption. Localized white matter effects appear limited to volumetric changes with microstructural organization unaffected.

## Significance Statement

Millions of children worldwide live in institutions under depriving conditions. Institutional deprivation has been linked to neurodevelopmental and mental health problems that often persist into adulthood. In this study, young adult adoptees who had experienced institutional deprivation as small children had smaller local volumes in multiple white matter tracts compared with nondeprived adoptees, based on diffusion tensor imaging. On the other hand, there were no deprivation-related difference in hindrance-modulated orientational anisotropy (HMOA) and fractional anisotropy (FA). This suggests that volumetric white matter alterations following deprivation persist into young adulthood, >20 years after the depriving conditions have ended, while microstructural white matter organization was either unaffected or had normalized by young adulthood.

## Introduction

Experience-dependent changes in white matter organization and structure are an important mechanism of neuroplasticity (Fields, 2005). White matter tracts connect brain regions and play a crucial role in efficient neural communication. Previous studies have demonstrated white matter alterations in tracts linked to learning and cognitive development following a range of different environmental experiences (Bengtsson et al., 2005; Sampaio-Baptista et al., 2013; Chorghay et al., 2018; Huber et al., 2018). Plasticity is particularly high during early postnatal development, when myelination on a micro-level, and white matter volume on a macro-level, increase substantially (Dubois et al., 2014; Gilmore et al., 2018). While this early plasticity is central to healthy development, it might also leave the brain particularly vulnerable to adverse experiences such as maltreatment during this period (Rutter et al., 2004). Human studies have linked early maltreatment to neurodevelopmental and mental health problems later in life (Gilbert et al., 2009), which may result from long-lasting exposure-induced changes in brain structure including white matter volume and microstructural organization (McCrory et al., 2010; Huang et al., 2012; Benedetti et al., 2014). However, most reports of such associations rely on observational and/or cross-sectional designs, many using retrospective accounts of maltreatment, which limit the authors’ ability to infer a causal relationship between maltreatment exposure and brain alterations (Sonuga-Barke et al., 2017). In fact, retrospective accounts of early adversity may capture processes largely separate from those measured by prospective studies (Danese, 2020). Where maltreatment occurred within biological families, interpretation is further complicated by the possibility of passive gene-environment correlations (Rutter, 2007).

Here, we report findings from a study focused on a specific population to address some of these limitations to strengthen casual inference. The English and Romanian Adoptees (ERA) study followed up children who had suffered extreme neglect in the Romanian institutions that existed toward the end of the Ceaușescu regime (i.e., a nonfamilial setting) for up to 43 months (i.e., a limited developmental period) before being adopted into well-functioning United Kingdom families, an abrupt change from a depriving to an enriching and nurturing environment (Rutter, 1998). The adoptees have recently been followed up in early adulthood and their brain structure has been assessed. Surface-based analyses showed that, compared with nondeprived United Kingdom adult adoptees, adoptees suffering institutional deprivation had substantially smaller total brain volume, effects equally marked for white and gray matter (Mackes et al., 2020). We also found localized deprivation-related variations in cortical gray matter volume in the inferior frontal, inferior temporal, and the medial prefrontal regions (Mackes et al., 2020).

In the present paper, we extend our analysis to white matter alterations using diffusion-weighted imaging (DWI) approaches to test whether deprivation is also associated with localized, tract-specific alterations in volume or microstructural organization. First, we used tensor-based morphometry (TBM) to create individual maps of local white matter volumetric deformations and identify the specific tracts implicated. The structural organization of affected tracts was then examined at the microstructural level using three metrics: hindrance-modulated orientational anisotropy (HMOA), fractional anisotropy (FA), and mean diffusivity (MD). HMOA is relatively novel approach that has been shown to be sensitive to changes in fiber diffusivity and microstructural organization and more likely to detect such changes compared with other DTI indices (Dell’Acqua et al., 2013).

Although no studies have yet examined adult white matter sequelae of early childhood institutional deprivation, there is evidence of an association between early deprivation and alterations to white matter structure in childhood and in adolescence. Smaller corpus callosum has been reported following institutional deprivation (Sheridan et al., 2012) and early maltreatment more generally (Teicher and Samson, 2016). Institutional deprivation has been linked to lower FA particularly in late-developing association fibers such as fornix, cingulum, uncinate, and inferior fronto-occipital fasciculi (IFOF; Eluvathingal et al., 2006; Hanson et al., 2013; Bick et al., 2015). Based on these prior studies, we hypothesized that childhood deprivation would be associated with long-term effects on adult white matter across multiple tracts seen in both localized smaller volume and alterations in microstructural organization.

Finally, we performed exploratory analyses to test whether changes in white matter tract volumes might be associated with neurodevelopmental outcomes shown to have persistent links with early deprivation in previous ERA follow-ups (Kennedy et al., 2016; Sonuga-Barke et al., 2017). These were symptoms of attention deficit hyperactivity disorder (ADHD), autism spectrum disorder (ASD), disinhibited social engagement (DSE), and intelligence quotient (IQ). We hypothesized that white matter tracts that have previously been implicated in neurodevelopmental conditions in samples drawn from the general population would also be predictive in this group (Cortese et al., 2013; van Ewijk et al., 2014; Wu et al., 2017).

## Materials and Methods

### Participants

A total of 165 Romanian adoptees and 52 nondeprived United Kingdom adoptees were included in the original ERA sample. Out of these, 81 Romanian and 23 United Kingdom adoptees took part in the ERA Brain Imaging Study (ERABIS). Eleven Romanian adoptees who had never been institutionalized were excluded from analysis because significantly higher variance in total brain volume indicated that their preadoptive environment might not be comparable (Mackes et al., 2020). Diffusion data of 11 Romanian adoptees and three United Kingdom adoptees were either not collected (because of scanner failure or meeting scanner exclusion criteria) or failed quality control checks. The final sample comprised 59 Romanian adoptees (35.8% of the original sample, 50.8% female, mean age = 25.3 years, age range = 23–28 years) and 20 United Kingdom adoptees (38.5% of the original sample, 40.0% female, mean age = 24.4 years, age range = 23–26 years). As Romanian adoptees typically entered the institutions in the first weeks of life, deprivation duration was measured as the age (in months) at which adoptees first entered the United Kingdom to join their adoptive families. Deprivation duration ranged between 3 and 41 months for the Romanian adoptees included in ERABIS. Diffusion data were collected at the KCL Centre for Neuroimaging Sciences at, London. All participants gave written informed consent to participate. They received a £100 Amazon shopping voucher as reimbursement for their time. Ethical approval was granted by the ethics committee of the University of Southampton and the Camberwell-St. Giles NHS Research Ethics Committee (Ethics No. 14/LO/0477).

### Procedure

We recruited and screened participants via mail and phone. The study protocol included two MRI scanning sessions, each lasting ∼1 h. Scans were typically performed on consecutive days. Before each session, participants were familiarized with the scanning environment in a mock scanner. In most cases, the diffusion scan was acquired during the first scanning session. The protocol also comprised neuropsychological testing and a questionnaire session. In total, the study took ∼8 h to complete.

Parents completed the Conners CBRS, SCQ, and the DSE interview questions during the previous follow-up study, the ERA Young Adult Follow-Up, when participants were aged between 22 and 26 years. These were used to explore the relationship between deprivation related white matter changes and adult neurodevelopmental symptoms of ADHD, ASD, and DSE which have been shown to be associated with deprivation in previous analyses (Sonuga-Barke et al., 2017).

### Measures

#### Subnutrition

Weight was recorded when children first arrived in the United Kingdom shortly after leaving institutions (Sonuga-Barke et al., 2008). Approximately 69% of the Romanian adoptees weighed <1.5 SDs below United Kingdom norms at that time. Weight at United Kingdom entry (in SD relative to age-matched and sex-matched United Kingdom norms) was used as a continuous variable as an index for subnutrition in the Romanian institutions.

#### ADHD symptoms

A total of 20 parent-rated items of the Conners Comprehensive Behaviour Rating Scales (Conners CBRS) were used to measure ADHD symptoms (Conners et al., 2011). The resulting symptom count (0–18 scale) represents the 18 DSM-5 ADHD symptoms. Items were adapted for young adults with permission from the copyright holders (Kennedy et al., 2016).

#### ASD symptoms

We measured ASD symptoms with 15 items of the parent-rated Social Communication Questionnaire (SCQ; 0–15 scale). Items were selected because they were considered developmentally appropriate for young adulthood (Rutter et al., 2003; Sonuga-Barke et al., 2017).

#### DSE symptoms

DSE symptoms were measured based on parents’ responses to interview questions which assessed the concepts of being “too friendly toward strangers,” showing “inappropriate intrusiveness,” and being “unaware of social boundaries.” Each symptom was rated as endorsed or not endorsed (0–3 scale; Sonuga-Barke et al., 2017).

#### IQ

IQ was assessed with the full version of the Wechsler Abbreviated Scale of Intelligence, Second Edition (WASI-II), which is a widely-used and reliable test of general intelligence (Wechsler, 2011).

### Diffusion data acquisition

All diffusion images were acquired on a General Electric MR750 3.0 Tesla scanner with a 12-channel head coil. Diffusion scans and their reverse phase images were acquired using spin-echo EPI (TR/TE = 11 650/70 ms, slice thickness = 2.0 mm, field of view = 256 mm, matrix size = 128 × 128, 72 slices). The reverse phase data included b0 images only and were collected to allow for distortion correction. Diffusion data were collected along 60 diffusion directions with a b-value of 1500 s/mm^2^.

### Diffusion data preprocessing

All images were controlled for quality. The preprocessing steps included de-noising (using Marchenko–Pastur distribution principal component analysis (Veraart et al., 2016), Gibbs ringing artifact correction (Kellner et al., 2016), and estimation of the susceptibility distortion field (Andersson et al., 2003). Distortion caused by head movement and eddy currents was corrected by using the Eddy toolbox in FSL including replacement of outliers (Andersson et al., 2016; Andersson and Sotiropoulos, 2016). Finally, the diffusion tensor was fitted using a nonlinear least squares algorithm in the DIFFCALC module, which is part of the TORTOISE software (Pierpaoli, 2018).

### Diffusion data analysis

Diffusion TBM (D-TBM; Sadeghi et al., 2018) uses log-transformed Jacobian deformation maps to measure local regional morphologic brain changes (Ashburner, 2007). Deformation maps are computed by diffeomorphically registering a participant’s MR image onto a group template. While most TBM approaches use T1-weighted structural images, these are not well suited to measure deformation in white matter regions because of signal homogeneity in these regions. D-TBM instead registers individual diffusion tensor images to a diffusion tensor template (Sadeghi et al., 2018). This approach is more sensitive in detecting morphometric alterations in white matter pathways compared with T1 map-based, or even FA map-based, TBM approaches (Hutchinson et al., 2018; Sadeghi et al., 2018).

Nonlinear registration of individual diffusion tensor maps to a group diffusion tensor template was performed using DR-TAMAS (Irfanoglu et al., 2016). The diffusion tensor template was based on a NatBrainLab dataset of 35 healthy adults aged between 20 and 30 years (www.natbrainlab.co.uk). A template independent of this study’s sample was chosen to prevent bias during group comparison. The transformation of each subject’s DT image to common space was based on an affine followed by a nonlinear warp. For each participant, a log-transformed map of the Jacobian determinant of the resulting warp field was computed. This calculation excluded the global affine term from the final Jacobian determinant to prevent local transformation values from representing any global scaling effects, as a previous ERA study showed that institutionalization was associated with smaller total white matter volume (Mackes et al., 2020). The Jacobian determinant encodes the scale of any volumetric expansion or contraction that occurred during registration. The resulting individual log-transformed maps therefore represent local brain volume differences relative to the template. Positive values indicate that an individual voxel is relatively bigger compared with the template, while negative values indicate the opposite.

### Tractography

Based on the D-TBM analysis, we discovered a number of tracts that showed alterations in local morphometry following institutional deprivation. We identified the following tracts that showed an overlap with the statistical clusters in the D-TBM analysis: left and right inferior longitudinal fasciculus (ILF), left and right IFOF, left superior longitudinal fasciculi (SLFs) I–III, left arcuate fasciculus, fornix, cingulum, left and right precentral and left and right postcentral corticospinal tracts. We performed manually guided dissections for these 14 tracts. The inclusion and exclusion ROIs for each tract have been detailed in previous papers (Catani and Thiebaut de Schotten, 2008; De Schotten et al., 2011; Howells et al., 2018).

First, spherical deconvolution modeling of the diffusion data was performed using StarTrack (https://www.mr-startrack.com) according to the following parameters: fiber response (α) = 1.5; number of iterations = 400; minimum peak amplitude = 0.0015; spatial regularization extent = 16. Deterministic tractography was then performed using the Euler algorithm and the following parameters: minimum HMOA threshold = 0.002; minimum angle threshold = 35 degrees; step size = 1 mm; minimum/maximum streamline length = 20/300 mm.

Tractography dissections, blind to the participants deprivation status, were then performed through the MegaTrack framework (Dell’Acqua et al., 2015). First, each participant’s tractogram was mapped to the space of the symmetric MNI template (https://www.bic.mni.mcgill.ca) by combining the tensor-based spatial transformations computed previously and a single transformation between the tensor template and the symmetric MNI template. The tractograms of all participants were then concatenated into a single group tractogram in which each streamline was assigned a unique subject-streamline ID (SSID). During concatenation, streamlines of the right hemisphere were symmetrically flipped into the left hemisphere to ensure that dissections were not biased to either hemisphere. This final tractogram was then manually dissected in TrackVis (http://trackvis.org). Using the assigned SSIDs, each subject’s dissected streamlines were selected in their native space without the need for additional spatial transformations, and macrostructural (volume) and microstructural diffusion metrics (HMOA, FA, and MD) were extracted.

For left SLFs II and III, diffusion metrics of two participants could not be extracted and were imputed based on diffusion metrics of the other tracts (using predictive mean matching with 200 iterations). Diffusion metrics were also imputed for left SLF I and left and right postcentral corticospinal tracts for one participant.

### Statistical analysis

#### Whole-brain analyses

For the whole-brain analyses we used the randomize toolbox in FSL (Winkler et al., 2014). We examined local white matter differences by investigating D-TBM log-Jacobian maps. We first tested for group differences between United Kingdom and Romanian adoptees using two-sample unpaired *t* tests. We then entered deprivation duration and subnutrition as predictors in separate regression models within the Romanian adoptees group. For all analyses, sex was entered as a covariate. A brain mask including only voxels with FA values ≥0.20 was applied to assure that analyses were restricted to white matter. For each analysis, 5000 nonparametric permutation tests were performed, threshold-free cluster enhancement (TFCE) was applied and the family-wise error rate (FWE) was controlled for to correct for multiple comparisons. Statistical maps were transformed into MNI space for labeling.

#### Region of interest analyses

Volume, HMOA, FA, and MD of each dissected tract showing significant volumetric effects at the whole-brain level were included as potential predictors in elastic net regression models, to test whether macrostructure and microstructure of these tracts were associated with institutionalization, deprivation duration or subnutrition experienced in the institutions. One elastic net regression model for each metric (volume, HMOA, FA, and MD) and deprivation measure (institutionalization, deprivation duration, subnutrition) was designed (12 models in total). Elastic net regression seeks to optimize the model by finding a balance between accuracy and parsimony (Zou and Hastie, 2003). It does so by adding a penalty term to the ordinary least squares regression model. Elastic net regression models are ideally suited for analyses such as ours, where predictors show collinearity (by shrinking estimates of collinear predictors) and not all predictors might be informative (by selecting informative variables and setting others to zero). In elastic net models, the penalty is controlled by α (α) and ranges between ridge (α = 0, penalization of norm 2) and lasso (α = 1, penalization of norm 1). The tuning parameter λ controls the overall strength of the penalty, with coefficients in the model shrinking more strongly as λ increases. We performed cross-validation to obtain optimal λ and α values. The initial range for α was between 0.2 and 1 in 0.1 steps. The initial range for λ was between 0.0001 and 10, increasing on a logarithmic scale with 300 values in total. For each level of α, the following calculations were performed: the dataset was randomly split into three folds with equal sample sizes. The model fit for each of the 300 λ values was computed with iteratively one of the folds omitted. The average mean squared error across folds was calculated for each λ. This cross-validation step was repeated 100 times. The λ with the lowest average mean squared error across all cross-validation iterations was selected for the final model at each level of α. Finally, the α with the lowest average mean squared error overall (and its corresponding λ value) were selected. For institutionalization, the model was specified as binomial, while it was Gaussian for deprivation duration and subnutrition.

#### Exploratory investigation of white matter tract volume and neurodevelopmental symptoms

We next tested whether volume of the selected tracts of interest predicted neurodevelopmental outcomes (IQ, ADHD, ASD, and DSE symptoms). We only included Romanian adoptees in these analyses to test whether white matter tracts predicted adult deprivation-related outcomes. We focused on volume, rather than HMOA, FA, and MD, as we were interested in deprivation-related associations only and previous analyses in this paper had shown no significant relationship between deprivation and the latter measures. Elastic net regressions were trained and cross-validated as described above. For IQ, the model was specified as Gaussian, while it was Poisson for ADHD, ASD, and DSE symptom counts.

## Results

### Whole-brain D-TBM

**Figure 1.**
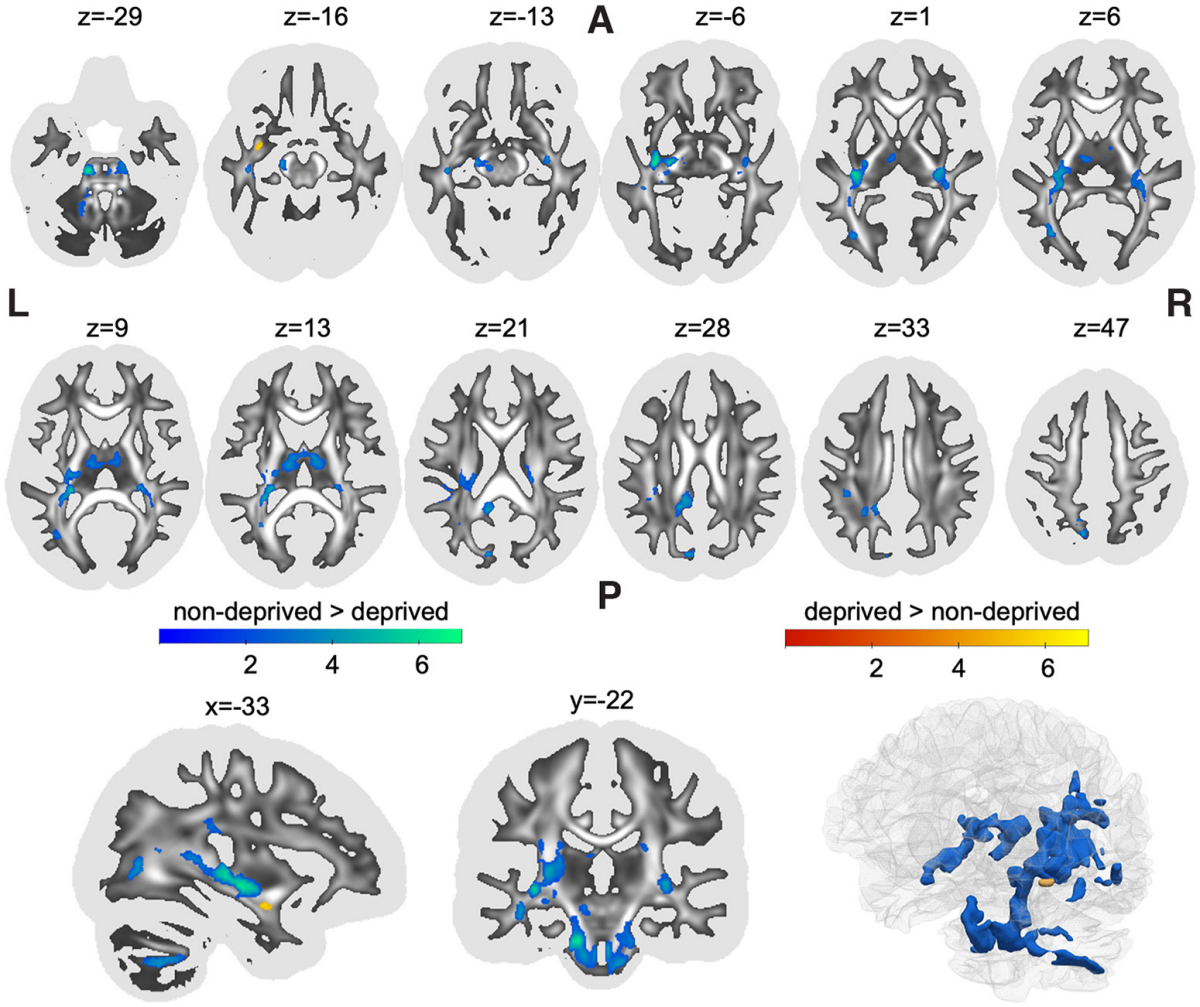
Clusters showing significant differences between deprived Romanian adoptees and nondeprived United Kingdom adoptees in the log-transformed Jacobian determinant of local white matter deformations. Statistical clusters that were smaller in volume in the deprived group (compared with the nondeprived group) are shown in blue, while regions that were bigger are shown in red. Statistical maps are shown as an overlay on a white matter map of the general population with whiter areas indicating higher FA. The FA map was also used as a binarized mask (threshold FA ≥ 0.20) for statistical analysis to assure that only white matter was included. The first two rows show axial slices. The last row shows a sagittal view, a coronal view and a 3D rendering. L, left; R, right; A, anterior; P, posterior; FA, fractional anisotropy. The figure was created using the software MRIcroGL (https://www.nitrc.org/projects/mricrogl/) and ParaView (https://www.paraview.org/).

Results were derived from whole-brain log-transformed maps of Jacobian determinants obtained when normalizing a participant’s brain to a standard template brain. Determinants therefore represent individual differences from template in local brain volume with higher values indicating larger local volume compared with the template. Institutional deprivation was associated with significantly smaller local white matter volume in multiple statistical clusters across the brain (Table 1). The biggest cluster spanned multiple white matter regions, starting caudally in the left middle cerebellar peduncle and bilateral brain stem (Fig. 1, *z* coordinate = −29), following the rostral line to intersect with the left iILF and IFOF (Fig. 1, *z* = 6). More superiorly, the cluster also overlapped with segments I, II, and III of the left SLF (SLF-I, SLF-II, SLF-III), as well as the long segment of the left arcuate fasciculus (Fig. 1, *z* = 21). The second biggest cluster included white matter of the bilateral thalamus as well as the fornix (Fig. 1, *z* = 13), spanning laterally to overlap with right ILF and IFOF (but not the SLF; Fig. 1, *z* = 6). The third cluster showed overlap with the posterior section of the cingulum (Fig. 1, *z* = 28). The remaining clusters were confined to small areas overlapping with left ILF, left IFOF, left cingulum and left SLF. Romanian adoptees showed larger local white matter volume in a small cluster that overlapped with the left uncinate fasciculus (Fig. 1, *z* = −16).

**Table 1:** Clusters showing significant differences between deprived Romanian adoptees and nondeprived United Kingdom adoptees in local white matter volumes (all *p_FWE_* < 0.05)

Cluster index	Cluster size (mm^3^)	Hemi-sphere	T value	Max voxel MNI coordinates (mm)			Annotation
				*x*	*y*	*z*	
Nondeprived > deprived							
1	12,460	L	6.43	−12	−23	−29	Brain stem
			6.28	−33	−17	−5	IFOF
			5.91	−33	−24	1	IFOF
			5.88	−33	−25	−1	IFOF
			5.48	−23	−17	−6	ILF
2	4760	R	4.58	33	−22	1	IFOF
			4.53	33	−22	−1	IFOF
			4.43	26	−33	9	IFOF
			4.28	29	−26	1	ILF
3	1791	L	4.56	−7	−47	17	Cingulum
			4.49	−12	−41	28	Cingulum
4	398	L	4.14	−34	−70	6	ILF
5	280	L	4.21	−9	−82	21	ILF
6	217	L	4.1	−41	−23	−13	ILF
7	140	L	4.78	−11	−67	47	ILF
8	117	L	3.8	−12	−57	51	Cingulum
9	98	L	3.37	−39	−37	33	SLF
10	97	L	3.86	−43	−35	−7	ILF
11	59	L	3.86	−31	−60	13	ILF
12	7	L	3.49	−37	−27	−16	ILF
Deprived > nondeprived							
1	82	L	6.00	−32	−4	−16	Uncinate
Deprivation duration							
No significant clusters							
Subnutrition							
No significant clusters							

L, left; R, right; IFOF, inferior fronto-occipital fasciculus; ILF, inferior longitudinal fasciculus. Threshold-free cluster enhancement (TFCE) was applied and all analyses were controlled for family-wise error rate (FWE).

Within the Romanian adoptees, there was no significant association between deprivation duration or subnutrition (as indexed by weight at first entry into the United Kingdom) and local white matter volume as measured here by log-transformed Jacobian deformation values (Table 1).

### Tract of interest analysis

Having identified deprivation-related white matter tract deformations at the whole-brain level, we investigated the macrostructure and microstructure of these tracts. We isolated the left and right ILF, left and right IFOF, fornix, left uncinate fasciculus, left arcuate fasciculus, left SLF-I, SLF-II, and SLF-III, as well as left and right corticospinal tracts (CST), divided into precentral (primary motor) and postcentral (somatosensory) CST. For these 14 tracts, we calculated individual tract volumes, HMOA, FA, and MD. For volume we report the final cross-validated elastic net model (i.e., with the lowest mean squared error, *R*^2^ = 0.152, *n *=* *79, α* *=* *0.4, *λ *=* *0.11). For this model, tractography-based results aligned with the findings of the D-TBM analysis: after regressing out total white matter volume, Romanian adoptees had lower white matter volume in the fornix (*b* = −0.160), left SLF-III (*b* = −0.147), left postcentral CST (*b* = −0.137), left SLF-I (*b* = −0.059), left SLF-II (*b* = −0.035), left IFOF (*b* = −0.022), right precentral CST (*b* = −0.012), and bigger white matter volumes of the left uncinate (*b *=* *0.324) and left precentral CST (*b *=* *0.218). White matter volumes were not associated with deprivation duration or subnutrition.

There was no association between HMOA, FA, and MD values and institutional deprivation (*n *= 79), duration of that deprivation (*n *= 59, Romanian adoptees only) nor subnutrition (*n *= 53 with available data) based on cross-validated elastic net models.

In exploratory cross-validated elastic net analyses, there was also no association between white matter tract volumes and IQ (*n *=* *54), symptoms of ADHD (*n *=* *47), ASD (*n *=* *44), or DSE (*n *=* *46).

## Discussion

This study provides evidence that early time-limited childhood institutional deprivation is associated with widespread localized alterations in white matter volume in young adulthood, even in individuals whose exposure to deprivation ended >20 years previously following their adoption into supportive and well-functioning households. These findings are in line with the hypothesis that severe early adverse experiences can have a long-lasting impact on brain development, that may persist even after many years of high-quality care (Rutter et al., 2004; Teicher and Samson, 2016). We found clusters with smaller local white matter volumes in previously deprived Romanian adoptees compared with nondeprived United Kingdom adoptees in multiple white matter tracts, most prominently the bilateral IFOF, bilateral iILF, fornix, left cingulum, left SLF, and bilateral corticospinal tract. There was one cluster in the left uncinate fasciculus that showed greater volume in Romanian adoptees. These findings were independent of previously observed differences in total white matter volume (Mackes et al., 2020). Given the greater number and more widespread distribution of localized effects compared with our gray matter findings (Mackes et al., 2020), it may be the case that deprivation-related alterations in white matter volume show higher region-specific variability compared with gray matter.

Long-ranging association fibers such as ILF, IFOF,SLF, and cingulum seemed particularly affected by deprivation suggesting they might be especially sensitive to early adversity because of their protracted postnatal development, with fronto-temporal connections being the last to mature (Dubois et al., 2014). In addition to association fibers, however, our study also found deformation in regions that contain projection fibers such as the thalamus, brain stem and cerebellum. Projection fibers connect these brain regions with the cortex and they develop relatively early compared with association fibers, with most dramatic increases in volume prenatally and in the first year postnatally (Geng et al., 2012; Dubois et al., 2014). This might indicate that profound deprivation very early in life, which in this study mostly started when infants were placed into institutions right after birth (Rutter et al., 2004), has a profound effect on white matter development affecting not only late-developing but also early-developing white matter tracts. The fact that tractography and D-TBM approaches both found the same pattern of morphologic changes is consistent with the hypothesis that deprivation negatively impacts the number of axons and fiber bundle cross-sectional size in the affected tracts (Raffelt et al., 2017).

Despite these volumetric effects, we found no evidence of deprivation-related alterations in microstructural organization of the affected tracts as indexed by HMOA, MD, and FA. One hypothesis is that underlying microstructural properties such as fiber density (Dell’Acqua et al., 2013) are less sensitive to institutional deprivation, potentially because fiber bundle size increases more substantially than fiber density in early childhood (Dimond et al., 2020). However, these findings appear to contradict the findings of previous studies of institutional deprivation effects on white matter microstructure in children. These studies reported changes in FA in the corpus callosum, external capsule, and retrolenticular internal capsule (Bick et al., 2015), as well as IFOF, ILF, corticospinal tract, cingulum, corona radiata and cerebellum, and thalamus (Hanson et al., 2013) and also uncinate fasciculus (Eluvathingal et al., 2006).

How can we explain these differences in findings? First, it is possible that prior findings linking deprivation to white matter microstructure are false positives resulting from, for instance, the use of exploratory a priori tract-of-interest analyses with insufficient correction for multiple testing. A second possibility is that the microstructural effects are small in scale compared with the morphologic effects and the current study was underpowered to detect them. A third possibility is that microstructural deprivation-related changes that present as lower FA during childhood and adolescence in previous studies may have resolved by young adulthood because of enrichment in the adoptive environment while macrostructural volumetric alterations as indexed by Jacobian deformation values are more persistent. Indeed, there is growing evidence that neurobiological systems can be recalibrated during adolescence opening up the possibility that some effects of early exposure to adversity can be corrected by exposure to positive adolescent experiences (Gunnar et al., 2019; Perry et al., 2022). That this could affect microstructural organization independent of volumes is supported by lifespan studies which show that developmental trajectories of tract volume and FA or MD are also only weakly correlated (Taki et al., 2013; Scherf et al., 2014).

We found scant evidence of alterations in the corpus callosum, a region that has previously been identified as sensitive to early maltreatment (McCrory et al., 2010; Teicher and Samson, 2016). One potential reason might be that changes in corpus callosum volume were present in childhood but have since normalized or recovered. This interpretation would be in line with findings from the Bucharest Early Intervention Project, which indicated a higher potential for recovery and plasticity in corpus callosum volume in children who had previously been institutionalized compared with gray matter regions (Sheridan et al., 2012).

What is the likely functional significance of these deprivation-related white matter alterations? We know that the affected tracts are involved in a wide range of cognitive processes important for daily functioning (Urbanski et al., 2008; Volle et al., 2008; Saur et al., 2010; Moritz-Gasser et al., 2013; Duffau et al., 2014; Budisavljevic et al., 2017; Herbet et al., 2017) and that our cohort present with cognitive impairments and continuing neurodevelopmental problems (Sonuga-Barke et al., 2017; Golm et al., 2021). However, in our exploratory analyses, we did not find an association between white matter tract volumes and neurodevelopmental outcomes within the Romanian adoptees group. As our brain imaging sample size was relatively small, it is possible that we were not powered enough to detect potentially more subtle effects.

We did not find any linear association between deprivation duration and local white matter deformation or DTI indices. This finding mirrors those reported on neuropsychological functioning in the same sample, where deficits were present regardless of the duration of the depriving experience (Golm et al., 2021). This suggests that extremely depriving conditions such as the ones encountered in Ceaușescu-era Romanian institutions can be associated with long-term changes in local white matter volume even if exposure lasted not more than a few months. Together with the aforementioned lack of association with neurodevelopmental symptoms, these findings also imply a nondeterministic relationship between white matter changes and neurodevelopmental outcomes where a step-wise increase was reported at six months of deprivation duration (Sonuga-Barke et al., 2017; Golm et al., 2021).

This study had many strengths. By harnessing the prospective longitudinal design of ERA, this study was able to overcome the limitations often associated with observational studies. For instance, adversity was limited to a precisely timed period, thereby allowing us to study the effects of adversity during the first months or years of life without subsequent deprivation in later development and without the risks of familial confounding (Sonuga-Barke et al., 2017). Second, by combining a D-TBM analysis with a tractography-based approach we were able to perform a whole-brain-based analysis while also being able to systematically investigate underlying macrostructure and microstructural alterations.

This study also had limitations. First, despite the strengths of the adoption study design, a lack of experimental manipulation limits our ability to draw definitive causal inferences about the effects of institutional deprivation on brain development. It is theoretically possible that Romanian adoptees show smaller local white matter volumes in specific areas compared with United Kingdom adoptees for other reasons than deprivation. A previous examination of underlying factors found no evidence that adoptees with smaller total brain volume were at increased genetic (as indexed by polygenic scores for intracranial volume) or prenatal (as indexed by birth weight) risk (Mackes et al., 2020). Consequently, we think it is unlikely that these factors are driving the differences in local white matter volume. This is also in line with the historic background of the ERA study, where institutionalization and timing of adoption were largely determined by external factors (i.e., the timing of the fall of the Ceauşescu regime). Second, as mentioned above, because of a relatively small sample size we might have had insufficient power to detect more subtle effects that might be associated with institutionalization, deprivation duration or subnutrition. Nevertheless the sample size was bigger than those of other studies investigating white matter (micro)structure in postinstitutionalized children (Hanson et al., 2013; Bick et al., 2015). Third, while the ERA study is longitudinal, the brain imaging aspect of the study was cross-sectional. Longitudinal imaging assessments are needed to identify how brain developmental trajectories are altered by institutionalization.

In conclusion, this study has shown that early institutional deprivation is associated with widespread localized white matter alterations in young adult adoptees, >20 years after they were removed from the institutions. Previously-institutionally deprived adoptees showed smaller volume in multiple white matter tracts compared with noninstitutionalized adoptees, while there were no group differences in microstructural organization. Our findings support the hypothesis that extreme adversity early in life can have a long-lasting impact on macrostructural white matter development despite later environmental enrichment.

## Synthesis

Reviewing Editor: Susanne Wolf, Charite University Hospital Decisions are customarily a result of the Reviewing Editor and the peer reviewers coming together and discussing their recommendations until a consensus is reached. When revisions are invited, a fact-based synthesis statement explaining their decision and outlining what is needed to prepare a revision will be listed below. The following reviewer(s) agreed to reveal their identity: NONE. Note: If this manuscript was transferred from JNeurosci and a decision was made to accept the manuscript without peer review, a brief statement to this effect will instead be what is listed below.

The current manuscript adds to a growing body of literature investigating the enduring effects of early life deprivation based on studies of children adopted from Romanian institutions by investigating the macro and microstructure of their white matter tracts.

Overall, the paper is well written and contains a nuanced discussion of the effects and lack of effects of early deprivation on white matter structure in the brain that the authors discovered.

I have very few comments to be addressed before publication:

1. line 111 “Here we report findings of a natural experiment...”

While I understand the point the authors are making, referring to the study design as a ‘natural experiment’ is awkward and seems inappropriate considering the experiences of the experimental group. I suggest changing this wording to “Here we report findings from a study focused on a specific population in order to address some of these limitations to strengthen casual inference.”

2. line 136 “Although no studies have examined adult white matter sequelae of early childhood deprivation, there is evidence of an association with white matter structure in childhood and adolescence.” This line would benefit from clarification, I suggest : “Although no studies have yet examined adult white matter sequelae of early childhood deprivation, there is evidence of an association between early deprivation and alterations to white matter structure in childhood and in adolescence.”

3. Line 386 : “We dissected...” This could be more accurately phrased as “We isolated” as dissected more closely implies a physical removal of the ROIs

## Author Response

We would like to express our gratitude to the reviewers for their helpful and positive comments. We have responded to each of their points below, and where appropriate highlight changes we have made to the manuscript in response to their comments.

We feel the manuscript is now stronger as a result of addressing the reviewers’ comments.

Thank you for further considering this manuscript for publication in eNeuro. We look forward to hearing from you in due course.

Yours sincerely, Nuria K. Mackes & Edmund Sonuga-Barke, on behalf of all co-authors.

Synthesis of Reviews

Synthesis Statement for Author (Required):

The current manuscript adds to a growing body of literature investigating the enduring effects of early life deprivation based on studies of children adopted from Romanian institutions by investigating the macro and microstructure of their white matter tracts.

Overall, the paper is well written and contains a nuanced discussion of the effects and lack of effects of early deprivation on white matter structure in the brain that the authors discovered.

I have very few comments to be addressed before publication:

Comment 1

Line 111 “Here we report findings of a natural experiment...”

While I understand the point the authors are making, referring to the study design as a ‘natural experiment’ is awkward and seems inappropriate considering the experiences of the experimental group. I suggest changing this wording to “Here we report findings from a study focused on a specific population in order to address some of these limitations to strengthen casual inference.”

Response

We thank the reviewer for asking for more caution with the wording. We have changed this sentence in line 111 as suggested.

Comment 2

Line 136 “Although no studies have examined adult white matter sequelae of early childhood deprivation, there is evidence of an association with white matter structure in childhood and adolescence.” This line would benefit from clarification, I suggest : "Although no studies have yet examined adult white matter sequelae of early childhood deprivation, there is evidence of an association between early deprivation and alterations to white matter structure in childhood and in adolescence.”

Response

This point is well taken. We have changed the sentence in line 136 accordingly.

Comment 3.

Line 386 : “We dissected...” This could be more accurately phrased as “We isolated” as dissected more closely implies a physical removal of the ROIs

Response

We thank the reviewer for raising this point. We have changed the sentence to “we isolated” as suggested.
